# Influence of Farming Conditions on the Rumen of Red Deer (*Cervus elaphus*)

**DOI:** 10.3390/ani9090601

**Published:** 2019-08-23

**Authors:** Federico Mason, Bartosz Fotschki, Alessia Di Rosso, Anna Korzekwa

**Affiliations:** 1 Department of Biodiversity Protection, Institute of Animal Reproduction and Food Research of Polish Academy of Sciences (IARFR PAS), 10-748 Olsztyn, Poland; 2 Department of Biological Function of Food, Institute of Animal Reproduction and Food Research of Polish Academy of Sciences (IARFR PAS), 10-748 Olsztyn, Poland

**Keywords:** red deer, rumen, nutrition, diet, rumen protozoa, rumen bacteria, rumen papillae

## Abstract

**Simple Summary:**

The diet offered to an animal in captivity may considerably differ from the natural one; this can affect the development of the digestive system, with connected influence on the health condition and welfare of the animal. Through a comparison of morphological and environmental characteristics of the rumen of red deer, we found out that, during autumn season, farmed deer have a limited choice of diet compared to wild ones living in the forest; this condition affected the morphology of the rumen wall and the composition of the rumen microbial population in the farmed animals. We recommend increasing the diversity of food offered to animals in captivity, with the aim of minimizing the negative effects of a poor variety of the diet on the digestive system.

**Abstract:**

The red deer is an intermediate feeder, showing a marked degree of forage selectivity, with seasonal morphological adaptations due to changes in food quality and availability. In captivity, deer have a limited choice of habitat and food, and we hypothesize that this condition affects the rumen environment. Rumen samples were collected from 20 farmed and 11 wild red deer in autumn 2018 in Poland, and analyzed for chemical composition, food residues, microbial population, and rumen papillation. Farmed deer had the highest *Campylobacter* spp., and total anaerobic bacteria, but lower *Clostridium* spp. Moreover, they showed a decrease in Diplodininae protozoa, and the presence of holotrichs that were absent in the wild animals. The rumen digesta of farmed animals had lower dry matter and acid detergent fiber than the wild ones. The analysis of food residues underlined the poor variety of the diet for animals in the farm. This apparently affected the papillation of the rumen, with animals of the farm having the shortest papillae of the *Atrium ruminis*. Overall, results suggest that red deer kept in farms, with a diet based mainly on grass, tree leaves, and some concentrate supplements, undergo a small modification of the rumen compared to the wild conspecifics.

## 1. Introduction

Captivity can be defined as the condition when an animal (either domestic or wild species) is held in confined space and is dependent on humans for provision of all its needs. This concept can relate, for example, to farms, private collections, breeding centers for reintroduction purposes, and zoos. As underlined in a review of 2005 [1], the captivity condition is characterized by factors with multiple potential effects on the behavior, physiology, and morphology of the animals, and one of these factor is represented by the food offered to the animals. In fact, as happens also in wild conditions, the diet has the potential to influence and modify the digestive system of the animal and, consequently, its physiology and health status. The modifications can apply both to the morphology (e.g., length of intestine, volume of stomach, tooth development) and the environment (e.g., microbial population, pH) of the entire digestive system.

According to Lin et al. [2], the morphology of the gastrointestinal tract of Formosan Reeves’s muntjacs (*Muntiacus reevesi micrurus*) kept in zoos was modified by the captive condition (in particular the diet offered to these animals); captive deer showed a different shape of ruminal papillae and a longer small intestine compared to wild ones. Also, Hofmann and Matern [3] reported that giraffes kept in a zoo showed marked differences in the gastric mucosa of the stomach and in the capacity of the stomach and cecum–colon compared to wild giraffes. Liukkonen-Anttila et al. [4] investigated morphological and physiological disparities between wild and captive capercaillies (*Tetrao urogallus*) fed on different diets; as result, wild birds had heavier gizzards, hearts, and livers, and longer small intestines and ceca than captive birds.

In 2018, a review on the ecology and evolution of the gut microbiome of nonhuman primates underlined the effect of the captive condition on different primate species [5]; in most of the studies examined by the authors, the diet offered to the captive animals had detrimental effects on the gut microbiome and, consequently, on the animal health. Eigeland et al. [6] in wild dugongs (*Dugong dugong*) detected dominant bacterial DNA bands that were not present in the captive animals; the authors suggested that this could be the effect of the different diet between wild and captive animals, and other captive factors such as the use of antibiotics.

The gut microbiota plays an important role in the biology of its host, for example, affecting its fitness (i.e., defense against pathogens, production of microbial metabolites [7]); this role is more prominent in ruminant species compared to others [8]. To maintain this important feature, the diet offered to the animals in captivity should be as similar as possible to the natural diet. 

The provision of a mismatched diet to wild ruminants in captivity can have a detrimental effect on the rumen microbial activity, and can lead to important gastrointestinal disorders such as ruminal acidosis [9]. According to Zenker et al. [10], it is very important to provide a feeding regime that prevents ruminal acidosis, as prophylactic health management in order to prevent the occurrence of other diseases correlated to this disorder typical in captive ruminants.

The red deer (*Cervus elaphus* L.) is a ruminant classified as an intermediate feeder and characterized by a marked degree of forage selectivity, with seasonal morphological adaptations due to changes in forage quality and food availability [11]. In Europe, in fact, its natural diet is composed of a wide range of plants (at least 145 different species) that, according to Gebert and Verheyden-Tixier [12], can be clustered into four principal groups: grass and sedges (29.6%), *Calluna* and *Vaccinium* (23.3%), leaves of deciduous trees and shrubs (10.2%), and conifers (8.8%). Red deer are common in parks and zoos, and they are also a farmed species. Deer farms are located in New Zealand, North America, Europe, and Asia, and are oriented to different purposes, such as meat production, stag farming to collect antlers, and reproduction farming to produce and sell breeding stocks [13]. Because of the captive conditions, deer have a limited choice of habitat and food, thereby reducing their intermediate feeder attitude. In captivity, the carrying capacity of the pasture can be different from the wild population density, and, to overcome these problems, captive deer are often offered conventional feedstuffs, such as hay, grains, and pellets [14,15]. 

Our hypothesis is that farmed deer are subjected to modifications of the digestive system because of the captive condition, in particular due to a diet different from the natural one. According to the presented background, this study aims to compare the rumen of farmed and wild red deer for morphological, chemical, and microbiological parameters.

## 2. Materials and Methods 

### 2.1. Ethical Statement

No animals were culled for the purpose of this study, and all samples were obtained postmortem.

### 2.2. Animals and Sampling

Rumen and blood samples were collected from a total of 31 adult red deer, during the autumn season in 2018 (October–November). Samples from 20 farmed deer were obtained at the “Ferma Jeleni Rudzie” farm, Warmian-Masurian Voivodeship, northeast Poland, from animals slaughtered for commercial purposes of the farm; animals are kept on a pasture and irregularly provided with supplements (such as grains, fruits, and pellets). Samples from 11 wild deer were obtained in three days of collection from animals shot by hunters during the hunting season in the Strzałowo Forestry, Warmian-Masurian Voivodeship, northeast Poland; this forest is characterized by the presence of pine and spruce groves with a mixture of birch, willow, aspen, maple, alder, and oak, allowing the animals to feed on a diet typical for this species in European forests [12].

For both groups, the same protocol was adopted: after opening the rumen, the content was manually mixed to obtain more representative samples, samples were collected for microbial population, and for chemical composition and food residue analysis, and the rumen wall was sampled for papillary size measurements. With the aim of evaluating the general health condition of the animals, and eventually excluding from the dataset those with suspected ongoing infection, blood for immunoglobulin (Ig) analyses was collected directly from the heart with a needleless syringe in tubes with anticoagulant (K2-EDTA, Cezamed, Olsztyn, Poland).

### 2.3. Immunoglobulins Analysis

After collection, blood samples were kept at 4 °C and, within 30 min, they were centrifuged (15 minutes at 1000× g at 2–8 °C) to separate the serum. Serum samples were then frozen at −20 °C until analysis. The samples were analyzed for IgG and IgM concentrations separately, with the use of commercial ELISA kits (Sheep IgG kit, catalog n. OKIA00186, Aviva System Biology; Prospecta Sp. z o.o., Warsaw, Poland, Bovine IgM kit, catalog n. DL-IgM-b, DLDEVELOP, Prospecta Sp. z o.o., Warsaw, Poland), according to the manufacturers’ protocols. Validation for IgG and IgM determinations was provided according to principles carried out for (i) precision as 20 repetitions of the same sample (exemplary deer plasma samples) determined for the same assay during five consecutive days, with each as a separate run, (ii) repeatability, (iii) reproducibility, and (iv) systematic error. Finally, the quality control was positive if the permissible total error did not exceed 10% and was calculated as the product of the coefficient of biological variation (1.65) × acceptable error of accuracy × permissible error of precision, according to the quality assessment provided in commercial, accredited diagnostic laboratories. Validation in these experiments was provided by one person. As a positive control, plasma samples collected from bovine peripheral blood earlier determined and with known concentration were added.

### 2.4. Bacteria Analysis

Samples of the mixed rumen content were collected and preserved in sterile Falcon tubes at stable temperature. On the same day of the collection (within six hours of collection), samples were incubated and analyzed for bacterial composition for specific groups, in accordance with the Polish Committee for Standardization (Polski Komitet Normalizacyjny—PKN). From each sample, 10-fold serial dilutions were prepared, according to the PN-EN ISO 6887-1 protocol. After incubation, grown colonies were counted, and the final results were expressed as mean colony-forming units (CFU)/g. Bacteria cultures were performed for the following bacteria: *Campylobacter* spp. (PN-EN ISO 6887-1), *Clostridium* spp. (PN-EN ISO 7931), *Escherichia coli* (PN-EN ISO 7251:2006), coliform bacteria (PN-EN ISO 4831:2007), and total anaerobic and aerobic bacteria (PN-EN ISO 4833).

### 2.5. Rumen Protozoa

Subsamples of the mixed rumen content were collected and strained through a plastic filter with 5-mm mesh to remove only the big particles of feed residue and to obtain a liquid sample. For each animal, a sample of filtered rumen liquid was added to the same volume of 10% formalin and was, therefore, preserved at room temperature for the microscope analysis. To estimate the protozoa concentration, the preserved samples were mixed with a glycerol–buffer–methyl green solution to stain protozoa nuclei [16], and with Lugol solution to stain skeletal plates [17]. The total cell count was performed with a Sedgewick Rafter counting chamber using an optical microscope (Olympus CX31, Olympus Polska sp. z.o.o, Warsaw, Poland) at 10× magnification [18]. Identification was done at a subfamily level, and groups were expressed as a percentage of the total.

### 2.6. Chemical Analyses

The mixed rumen content was sampled for a total of 500 g, and later frozen at −20 °C. Aliquots of frozen rumen content were later analyzed for dry matter (DM), ash, and crude protein (CP) contents according to AOAC protocols (methods 930.15, 942.05, and 984.13, respectively [19]), for lipid content according to Folch method [20], and for neutral detergent fiber (NDF) and acid detergent fiber (ADF) contents according to van Soest et al. [21].

The pH of samples was recorded after thawing by means of a pH-meter (LAB 855, SI Analytics GmbH, Weilheim, Germany).

### 2.7. Food Composition

About 100 g of frozen rumen content collected for the chemical analyses was checked for food composition. After thawing, the sample was washed with tap water in a 1-mm wire mesh strainer, then squeezed to remove excess of water, and spread on a table. Food residues were macroscopically identified, and grouped into the following categories: “herbaceous plants”, “leaves of deciduous trees”, “fruits and seeds”, “bark, twigs, and gems”, “catkins of deciduous trees”, “needles of coniferous trees”, “*Calluna* and *Vaccinium*”, “ferns”, and “lichens, mosses, and mushrooms”, plus two additional categories (“others” and “parasites”). No quantification of the different categories was made. Results were expressed as prevalence, which is the number of animals showing the elements of the category as a function of the total animals of the same group (farmed or wild).

### 2.8. Rumen Papillary Size

After collecting the samples for the previous described analyses, the rumen was emptied and a rectangular sample of rumen wall was taken from the dorsal area, the ventral area, and the *Atrium ruminis* [22]; wall samples were preserved in 10% formalin. For the papillary analysis, a subsample of 2 × 2 cm was taken from the central area of the preserved samples, and used to count the number, and measure the length and the width of the papillae, according to Lentle et al. [23] and Mathiesen et al. [24]. 

The “surface enlargement factor” (SEF) was calculated as described by Hofmann et al. [25].

SEF = (((LP × WP × 2) × NP) + 100)/100,

Where LP is the mean papilla length (mm), WP is the mean papilla width (mm), and NP is the mean number of papillae per cm^2^.

### 2.9. Statistics and Calculations

Statistical analyses were performed with the R software [26], “dplyr” package [27]. Before analysis, the data were analyzed for normal distribution with the Shapiro–Wilk test. Successively, datasets were analyzed with a parametric test (Welch *t*-test) or with a non-parametric test (unpaired two-sample Wilcoxon test) for the “environment” effect.

Only bacteria count data were converted to log_10_ before the analysis for comparison purposes. All tests were two-tailed, and the significance level was set at *p* < 0.05.

## 3. Results

### 3.1. Animals and Sampling

Due to logistic limits, a lower number of wild deer than planned was sampled (e.g., because of availability during hunting sessions); this led to a different sample size between the two groups.

### 3.2. Immunoglobulin Analysis

Immunological analysis showed one farmed animal being negative for IgG and positive for IgM (52.17 ng/mL); therefore, we decided to exclude it from the dataset for suspected ongoing infection. All the other animals were positive for IgG and negative for IgM in the serum; no statistically significant differences were found between the two groups for IgG values (197.55 ± 22.03 vs. 158.96 ± 61.09 μg/mL, respectively, for farmed and wild animals; *p* = 0.577).

### 3.3. Bacteria Analysis

Bacteria abundances are presented in Table 1. Farmed animals, compared to wild animals, had higher *Campylobacter* spp. (3.59 vs. 1.37 log_10_ CFU∙g^−1^, respectively; *p* < 0.001) and total anaerobic bacteria (6.49 vs. 5.68 log_10_ CFU∙g^−1^, respectively; *p* < 0.001), but lower *Clostridium* spp. (3.62 vs. 5.89 log_10_ CFU∙g^−1^, respectively; *p* < 0.001). *Escherichia coli*, coliforms, and aerobic bacteria were not statistically different between the two groups.

### 3.4. Rumen Protozoa

**Figure 1:**
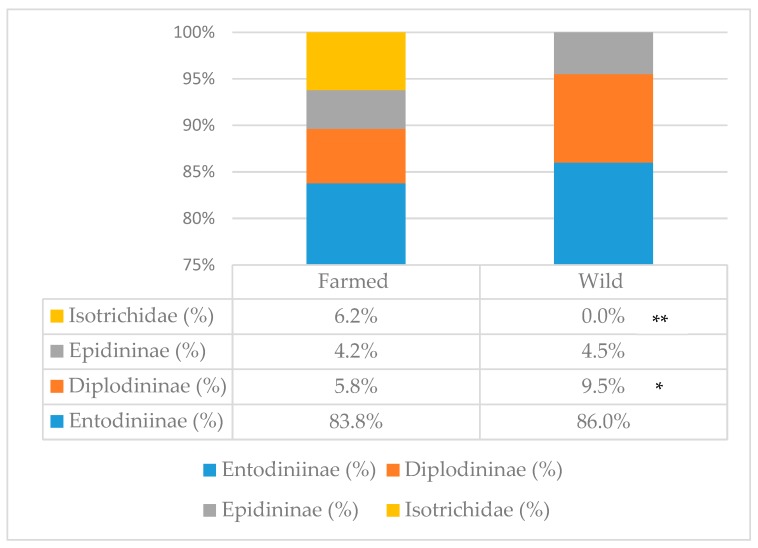
Protozoa subfamily distribution in the rumen of farmed and wild red deer. Statistically significant differences are denoted as * (*p* < 0.05) or ** (*p* < 0.001). Farmed: *n* = 19; wild: *n* = 7.

Total protozoa count (Table 1) was not affected by environment; however, the percentage of the different subfamilies was (Figure 1). In farmed animals, the presence of Isotrichidae protozoa was notable (6.2% of the total population), while they were totally absent in wild animals; moreover, farmed animals showed a lower percentage of Diplodininae protozoa compared to wild ones (5.8% vs. 9.5%, respectively; *p* < 0.05). Epidininae and Entodiniinae protozoa percentages did not differ between the two groups, with the latter one being the most abundant (84.9% on average).

### 3.5. Chemical Analysis

Table 2 shows the results of the chemical analyses on the rumen contents. Farmed deer, compared to wild deer, had rumen digesta lower in DM content (127.69 vs. 142.42 g/kg, respectively; *p* < 0.05) and in ADF percentage (283 vs. 330 g/kg DM, respectively; *p* < 0.05). No statistically significant difference was found for CP and NDF contents; furthermore, ash and fat contents showed tendencies (*p* < 0.10), being the lowest for farmed animals. The rumen content of farmed animals resulted more acidic than that of wild animals (6.14 vs. 6.51, respectively; *p* < 0.05).

### 3.6. Food Composition

**Figure 2:**
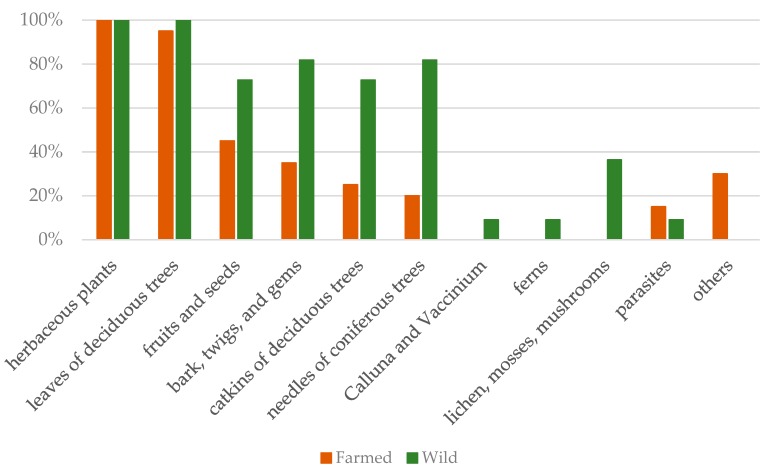
Food category prevalence in the rumen of farmed and wild red deer, calculated as the percentage of animals presenting that food category as a function of the total sample size (farmed: *n* = 19; wild: *n* = 11).

The analysis of food residues allowed distinguishing some plant categories typical only of the wild animals, and not present in the farmed ones. Figure 2 shows the prevalence of each category for farmed and wild deer. For both groups, the highest prevalence was for herbaceous plants (100% for both groups) and leaves of deciduous trees (100% for wild animals, and 95% for farmed animals); moreover, farmed deer shared (in much lower prevalence) with the wild ones four other categories (fruits and seeds; bark, twigs, and gems; catkins of deciduous trees; needles of coniferous trees). In the rumen content of wild deer, four further categories were exclusive (*Calluna* and *Vaccinium*; ferns; lichens, mosses, and mushrooms; roots). The “other” category included non-food material (like stones or plastic pieces), and the “parasites” category represented the presence of the adult stage of flatworms belonging to the Paramphistomidae family.

### 3.7. Rumen Papillary Size

Measurements done on the rumen wall are presented in Table 3. Farmed animals had on average shorter papillae of the *Atrium ruminis* wall compared to wild ones (6.56 vs. 9.28 mm, respectively; *p* < 0.05); moreover, there was a tendency (*p* = 0.0570) for the papillae of the dorsal wall to be shorter in famed animals than in wild ones (2.87 vs. 3.73 mm, respectively). All other measurements on the rumen papillae did not differ between the two groups. Generally, papillae of the *Atrium ruminis* were bigger than those of ventral and dorsal walls (*p* < 0.001).

## 4. Discussion

The present study aimed to compare farmed and wild red deer for rumen characteristics. One of the main factors that changes because of the captive condition is the diet offered to the animals [1]. It is known that a shift in diet influences the rumen in its environment (e.g., microbial population) and in its morphology (e.g., degree of papillation and volume capacity), and can lead to health and nutritional problems. Mathiesen et al. [24] showed in reindeer that a variation in diet characterized by different rates of fermentation had an influence on the rumen papillae development.

The rumen of both domestic and wild animals is inhabited by microorganisms belonging to different taxa; most of them are commensals or symbionts, but few of them are potentially pathogens (both for the ruminant and for the human). Some authors investigated the occurrence of *Escherichia coli* [28] and *Campylobacter* spp. [29] in the rumen of domestic cattle, as a potential reservoir for bacteria contamination during slaughter. Venison consumption by humans comes both from wild and farmed deer. While shooting the animals, a damage of the digestive organs can increase the risk of meat contamination; in relation to this, in the present study, the microbiological analyses focused on the abundance of some potentially pathogenic bacteria present in the rumen of farmed and wild red deer. Farmed deer had on average higher abundance of *Campylobacter* spp., but lower *Clostridium* spp., compared to wild animals. Moreover, they showed the highest prevalence of coliforms and *Campylobacter* spp. in the rumen. Coliforms are not usually considered as true rumen microorganisms [30], while *Campylobacter* spp. can be detected in the rumen, but usually in lower concentration than the lower gut [29]. *Clostridium* spp. strains (including the pathogenic *C. perfringens*) are often found in the rumen, although they are not considered as predominant bacteria [31] and were previously reported also in the red deer [32]. Our data confirm this with 100% prevalence for both wild and farmed deer. Most *Clostridium* species are able to degrade cellulose, whereas some of them have also amylolytic or proteolytic activity [33]; in the present study, the higher concentration found in the rumen of wild deer might be related to the higher ADF content of the diet, compared to farmed animals. *Escherichia coli* was detected in both groups, with higher prevalence in wild animals, but with a bacteria concentration not statistically different from the farmed animals. The method used for the analysis did not allow distinguishing the species of *Clostridium* and *Campylobacter*, nor the serotype of *E. coli*; therefore, we can only say that the detected bacteria are potentially pathogens. Nevertheless, based on our results, we underline the importance of high hygiene levels and caution to not damage the rumen during the slaughter of both farmed and wild deer.

Bacterial population in the rumen of domestic cattle occurs at a concentration of 10^10–11^ cells/g of rumen content [34], while other authors reported a total count of 10^9^ in red deer [35,36]. Our results show a concentration of anaerobic and aerobic bacteria in a range of 10^5–6^ cells/g. We cannot consider the sum of the two categories as an indication of total bacteria count, because the anaerobic analysis estimates also include the facultative anaerobes; however, the concentration of anaerobic bacteria in our samples is quite low, and this might be due to oxygen contamination during the sampling procedure. 

Rumen protozoa represent up to 50% of the viable biomass in the rumen, are able to engulf bacteria and feed particles, and can digest carbohydrates, protein, and fats [34]. In our study, farmed deer did not differ for total rumen protozoa concentration from their wild conspecifics; however, they did for the community composition. The average total number of rumen protozoa (3.29 × 10^6^/mL of rumen fluid) in this study was higher than that reported by other studies on red deer [35,37,38], but similar to the number reported by Hobson et al. [36]. Rumen fluid of farmed animals was characterized by a lower percentage of protozoa belonging to the Diplodininae subfamily, and by the presence of protozoa of the Isotrichidae family (or holotrichs), totally absent in wild animals; Entodiniinae and Epidininae protozoa were present at a similar concentration in the two groups, with the Entodininae subfamily having the highest concentration. According to the literature, there is no consistency in the rumen protozoa composition of the red deer. Prins and Geelen [37] and Dehority [39] reported the presence of protozoa belonging to the subfamilies of Entodiniinae, Diplodininae, Epidininae, and the family Isotrichidae in confined and domesticated red deer, while Gnat et al. [35] did not find any Isotrichidae protozoa in wild red deer. This is consistent with our results, where the holotrich protozoa were present only in farmed animals. According to the review of Williams [40], chemical and physical characteristics of the diet, the feeding frequency, and rumen retention rate impact the protozoa population composition; moreover, holotrich protozoa are more present in domesticated than wild animals, and, in European ruminants, they occur more in grazers than in browsers. However, from the more recent review of Clauss et al. [41] on the effect of feeding type and body mass of wild ruminants on the rumen protozoa population, Isotrichidae protozoa are not correlated to the feeding type, but to the body mass (and, therefore, to the volume of the rumen); according to the authors, cattle-type animals offer a better rumen niche for holotrich protozoa. From the same review, it appears that the general tendency of intense feeding for domestic animals relates to lower numbers of Diplodininae, and higher number of Isotrichidae protozoa; this could be consistent with our findings, if we consider the group of farmed deer as “domestic”.

The chemical composition of the rumen content is strictly related to the diet, and we expected differences between the two groups of red deer. The DM of the rumen contents had an average value of 135.06 g/kg. No comparison values were found in the literature for the rumen DM content of red deer; however, Popović et al. [42] reported higher values for roe deer in the autumn. According to the authors, the highest value of DM for roe deer was due to the ingestion of acorns and grains. Red deer, as an intermediate feeder, tends to feed more on grasses and forbs, thus lowering the DM content of the digesta compared to the roe deer (which is classified as a browser or concentrate selector). In our study, wild red deer had higher DM and ADF percentages of the rumen content than the farmed deer; this might be due to the presence in the diet of more lignified materials such as bark and twigs. The mean value of CP was 237 g/kg DM, being higher than the percentage of protein suggested for deer diet (12–16% CP [43]). The reason is that the value measured in the digesta includes not only diet protein but also nitrogen from rumen microorganisms, from saliva, and from ammonia. The rumen content of farmed animals had lower pH values than the wild ones, both values remaining in the optimum range for microbial activity [34].

From the review of Gebert and Verheyden-Tixer [12], in the types of habitat that also characterize the Strzałowo Forestry, the red deer would feed on *Calluna* and *Vaccinium*, and coniferous browse in the areas of mixed coniferous forest, on fruit, deciduous leaves, shrubs, twigs, and bark in the areas of mixed deciduous forest, and on grass and sedges in all habitats. In the present investigation, the analysis of food residues in the rumen showed that both groups of animals fed on herbaceous plants and deciduous leaves. More than 70% of wild deer were also feeding on “fruits and seeds”, “bark, twigs, and gems”, “catkins of deciduous trees”, and “coniferous needles”. Farmed animals had the lowest prevalence for these categories, probably for the poorer availability of these foods at the farm; however, it should be noticed that the category “fruits and seeds” consisted mainly of apples and conkers of horse-chestnut, most probably provided by the farmers. Elements typical of forest habitat, like ferns, lichens, mosses, mushrooms, and shrubs, were found only in the rumen content of wild deer. While analyzing the rumen content for food residues, we also took note of the presence of rumen parasites (Paramphistomidae family), reporting them for either group (note that farmed animals are treated with anti-parasitics, and had higher prevalence than wild ones). This was not a purpose of the present study; however, we speculate that this result might be due to resistance of the adult flukes to the most common anti-parasitics, and the lack in the diet of plants containing chemical compounds with anti-parasitic activity [44] (as in the natural diet of the red deer).

The rumen papilla development is connected to the quality and quantity of the different ingredients of the diet, with morphological adaptation influenced by the nutritional content and the physical form of the diet [16,23]. The measures taken on the rumen papillae showed results comparable to those reported by other authors [36,45]. Overall, papillae of the dorsal and ventral walls were smaller than those of the *Atrium ruminis*, and this is expected considering that the latter is the main absorptive area in the rumen. The previously described differences in the composition of the diet of the two groups influenced the papillation level of this area; in fact, farmed animals had the smallest papillae of the *Atrium ruminis* wall. These results agree with the work of Lentle et al. [23], who found a significant effect of the habitat on rumen papillae, with farmed deer having significantly smaller papillary size than wild ones; the authors explained this effect with limited choice of food by the farmed animals, which had a diet based on grass and, therefore, a lower fermentation rate. In our study, almost all deer had a diet based on grass and deciduous leaves, but very few farmed animals also included other food categories in the diet. The NDF content did not differ between the two groups; however, the diet based on grass, which is characterized by a slow fermentation rate and a different effect of the physical form of the food, might be the explanation for the reduced size of the rumen papillae in the *Atrium ruminis* for farmed deer.

## 5. Conclusions

Results of the present investigation underline how the red deer is able to adapt the rumen to the farming condition. Farmed animals feeding mainly on grass pasture showed changes in the rumen microbial composition, and were subjected to morphological adaptation of the rumen papillae. The poor offer of plants at the farm caused a reduced variety of the diet, with animals feeding predominantly on herbaceous plants and deciduous leaves; however, it seems that, when possible, the farmed animals added food elements typical of the natural environment to the diet (as indicated by the low prevalence of four food categories in common with the wild deer, without considering “herbaceous plants” and “deciduous leaves”). Although, from the nutritional point of view, the diet at the farm seemed adequate for the red deer, its low variety may have a detrimental result on all the positive effects coming from the provision of secondary plant compounds (e.g., tannins) typical of the natural diet, such as control of pathogenic bacteria and parasites. To conclude, we suggest that the diet offered to captive red deer should be as similar as possible to the natural diet, characterized by a good variety of plants that allows the animals to express their selective attitude according to their nutritional needs. 

## Figures and Tables

**Table 1:** Bacteria abundances and total protozoa count in rumen of farmed and wild red deer.

Microorganisms	Concentration ^1^ (mean ± SD)		Statistics		Prevalence ^2^	
	Environment				Environment	
	Farmed (*n* = 19)	Wild (*n* = 11)	W	*p*-Value	Farmed (*n* = 19)	Wild (*n* = 11)
*Campylobacter* spp.	3.58 ± 0.09 (4.67)	1.37 ± 1.40 (0.38)	205	<0.001	100	54.5
*Clostridium* spp.	3.63 ± 0.27 (7.34)	5.89 ± 0.29 (919.4)	0	<0.001	100	100
Coliforms	3.29 ± 0.40 (4.01)	2.42 ± 1.63 (3.96)	134.5	0.2036	100	72.7
*Escherichia coli*	0.96 ± 1.75 (0.30)	1.74 ± 1.39 (0.40)	66.5	0.078	36.8	63.6
Anaerobic bacteria	6.48 ± 0.09 (3606)	5.68 ± 0.46 (783.5)	192	<0.001		
Aerobic bacteria	6.19 ± 0.50 (3346)	5.93 ± 0.40 (1205)	145	0.085		
Total protozoa ^3^	2.62 ± 1.59	3.97 ± 2.07	38	0.1055		

^1^ Values expressed as log_10_ colony-forming units (CFU)∙g^−1^; in brackets, values in *n* × 10^3^ CFU∙g^−1^.^2^ Calculated as percentage of animals positive for that bacteria type in the total sample size (*n*).^3^ Values expressed as *n* × 10 ^6^/mL; forest: *n* = 7.

**Table 2:** Chemical composition and pH of the rumen content of farmed and wild red deer. DM—dry matter; CP—crude protein; NDF—neutral detergent fiber; ADF—acid detergent fiber.

Variable ^1^	Environment		Statistics	
	Farmed (*n* = 19)	Wild (*n* = 11)	*t*	*p*-Value
DM (g/kg)	127.69 ± 17.30	142.42 ± 17.70	−2.2295	<0.05
Ash (g/kg DM)	152 ± 32	132 ± 18	1.8533	0.0744
CP (g/kg DM)	237 ± 59	237 ± 21	0.018158	0.9857
Fat (g/kg DM)	184 ± 34	167 ± 14	1.9366	0.0638
NDF (g/kg DM)	542 ± 51	520 ± 29	1.2563	0.2198
ADF (g/kg DM)	283 ± 61	330 ± 29	−2.7595	<0.05
pH	6.14 ± 0.35	6.51 ± 0.52	−2.3413	<0.05

^1^ Values expressed as means ± SD.

**Table 3:** Rumen papillae measurements ^1^ in three different areas of the rumen wall of farmed and wild red deer.

Variable ^2^	Ventral Wall				Dorsal Wall				*Atrium Ruminis* Wall			
	Environment		Statistics		Environment		Statistics		Environment		Statistics	
	Farmed ^3^	Wild ^4^	W	*p*-Value	Farmed	Wild	W	*p*-Value	Farmed	Wild	W	*p*-Value
LP	2.86 ± 0.66	3.57 ± 1.19	21	0.1877	2.87 ± 1.27	3.73 ± 0.98	15	0.0570	6.56 ± 1.66	9.28 ± 2.61	13	<0.05
WP	1.16 ± 0.21	1.24 ± 0.24	24	0.3055	1.30 ± 0.40	1.20 ± 0.35	41	0.5914	1.96 ± 0.32	1.75 ± 0.46	48.5	0.2043
NP	60.45 ± 19.51	62.86 ± 16.40	33	0.8836	57.43 ± 17.48	70.50 ± 17.43	21	0.1874	54.60 ± 12.62	58.07 ± 23.23	36.5	0.9222
SEF	4.99 ± 1.72	6.53 ± 2.72	23	0.2617	5.81 ± 3.90	7.34 ± 3.18	19	0.1874	14.73 ± 4.79	20.12 ± 12.61	26	0.4068

^1^ Values are expressed as means ± SD.^2^ LP: papilla length (mm); WP: papilla width (mm); NP: number of papillae per cm^2^; SEF: surface enlargement factor.^3^ *n* = 19.^4^ *n* = 11.
